# Human hippocampal processing of environmental novelty during spatial navigation

**DOI:** 10.1002/hipo.22264

**Published:** 2014-03-06

**Authors:** Raphael Kaplan, Aidan J Horner, Peter A Bandettini, Christian F Doeller, Neil Burgess

**Affiliations:** 1 NIMH-UCL Joint Graduate Partnership Program in NeuroscienceBethesda, Maryland; 2 UCL Institute of Cognitive Neuroscience, University College LondonUnited Kingdom; 3 UCL Institute of Neurology, University College LondonUnited Kingdom; 4 Section on Functional Imaging Methods, Laboratory of Brain and Cognition, National Institute of Mental HealthBethesda, Maryland; 5 Radboud University Nijmegen, Donders Institute for Brain, Cognition and BehaviourThe Netherlands

**Keywords:** amygdala, fMRI, MTL, content, context

## Abstract

The detection and processing of novel information encountered as we explore our environment is crucial for learning and adaptive behavior. The human hippocampus has been strongly implicated in laboratory tests of novelty detection and episodic memory, but has been less well studied during more ethological tasks such as spatial navigation, typically used in animals. We examined fMRI BOLD activity as a function of environmental and object novelty as humans performed an object-location virtual navigation task. We found greater BOLD response to novel relative to familiar environments in the hippocampus and adjacent parahippocampal gyrus. Object novelty was associated with increased activity in the posterior parahippocampal/fusiform gyrus and anterior hippocampus extending into the amygdala and superior temporal sulcus. Importantly, whilst mid-posterior hippocampus was more sensitive to environmental novelty than object novelty, the anterior hippocampus responded similarly to both forms of novelty. Amygdala activity showed an increase for novel objects that decreased linearly over the learning phase. By investigating how participants learn and use different forms of information during spatial navigation, we found that medial temporal lobe (MTL) activity reflects both the novelty of the environment and of the objects located within it. This novelty processing is likely supported by distinct, but partially overlapping, sets of regions within the MTL.

## INTRODUCTION

When exploring our environment, we must react to changes in our overall surroundings, but also simultaneously detect the novel content located within our environment. How the brain processes these different forms of novelty is not fully understood. A prime candidate for a role in novelty detection is the hippocampus (Knight, 1996; Stern et al., 1996; Strange et al., 2005a,b; Kumaran and Maguire, 2006; for reviews see Martin, 1999; Ranganath and Rainer, 2003; Nyberg et al., 2005), an area normally associated with human mnemonic function (e.g., Scoville and Milner, 1957; see Eichenbaum and Cohen, 2001; Squire et al., 2004 for review). However, it is unclear from human and rodent models of the hippocampal system whether the hippocampus preferentially encodes novel content or contexts. Some studies in humans have found that the hippocampus responds to individual novel stimuli (e.g., Knight, 1996; Strange et al., 2005a,b; Daselaar et al., 2006), while others have reported novel pictures/contexts/object pairings eliciting hippocampal activation (Stern et al., 1996; Kohler et al., 2005; Kumaran and Maguire, 2006).

Human intracranial EEG and fMRI data and studies in animal models have also implicated other medial temporal lobe (MTL) regions with mixed results. The perirhinal cortex was found to respond to novel objects and also stimuli pairings in humans (Pihlajamaki et al., 2003, 2004) and novel object identification in nonhuman primates and rodents (Buckley and Gaffan, 1998; for review see Brown and Aggleton, 2001). Previous studies have found pre-rhinal/parahippocampal cortex responses to novel contexts across species (Bussey et al., 1999; Epstein et al., 1999; Preston et al., 2010), but one study did find parahippocampal cortex responses to object novelty (Pihlajamaki et al., 2004) that could be related to its hypothesized role in retrieving individual representations of a context (see Eichenbaum et al., 2007 for review). Although the amygdala is most commonly activated in fMRI paradigms using affective and reward-related stimuli (Phelps, 2006; Seymour and Dolan, 2008; Adolphs, 2010), studies have also implicated the amygdala in detection of novel objects both in humans (Halgren et al., 1980; Fried et al., 1997; Rutishauser et al., 2010) and rodents (Moses et al., 2002; Sheth et al., 2008; Farovik et al., 2011). Consequently, it is unclear how different MTL structures might process novel objects and environments during a naturalistic spatial learning task.

**Figure 1:**
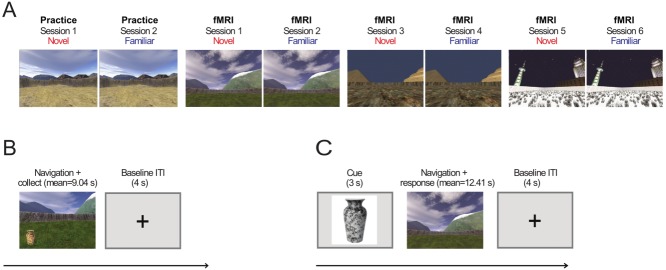
Experimental Structure. A: Experimental environments shown from the participants' (first-person) perspective. Four different environments are presented in eight experimental sessions. The first two sessions (always the desert environments) provided practice outside of the scanner. Sessions 3–8 contained three novel-familiar environment repetitions with environment order, counterbalanced across participants. B: Learning phase trial structure. During learning trials, participants use a button box to navigate and “collect” three novel and three familiar (previously presented) objects (vase shown as example) four times each (a total of 24 trials per session). C: The object replacement phase, trial structure. After being cued for 3 s with a picture of an object that had been collected in the learning phase of the current session, participants were placed back in the environment and had to navigate to where they thought the object (object replacement) had been located during that learning period. [Color figure can be viewed in the online issue, which is available at wileyonlinelibrary.com.]

We examined the effects of environmental and object novelty on fMRI activity during a virtual spatial memory paradigm, similar to tasks used with rodents (see also Doeller et al. 2008, 2010; Kaplan et al., 2012), see Figure 1. Participants used a button box to navigate a first person perspective within virtual environments. Within each session, they learned the locations of six objects encountered within the environment, over four trials per object. In the replacement phase of the experiment, they were then cued by a picture of an object, replaced in the environment and had to navigate to the object's location, for one trial per object. Each session in a novel environment was followed by a session in the same (now familiar) environment. Three of the objects encountered in a session were new to that session and three had been encountered before in a different environment (see Fig. 1).

## MATERIALS AND METHODS

### Participants

Twenty male participants (mean age years = 23.9; SD = 3.7 years; range = 18–33) gave written consent and were compensated for performing the experimental task, as approved by the local Research Ethics Committee at University College London. All participants were right-handed with normal or corrected-to-normal vision and reported to be in good health with no prior history of neurological disease. Two participants were excluded from the fMRI analyses because of scanner malfunction.

### Stimuli, Task, and Trial Structure

As in Kaplan et al., 2012, the experiment was composed of eight sessions. The first two sessions were practice sessions in the same virtual desert environment, conducted on a laptop outside the MRI scanner. The participants familiarized themselves with the environment by navigating around and then collecting objects in the environment by running into them and then being tested on their previous location. These practice sessions lasted for ∼2–3 min.

During the fMRI sessions, an individual trial consisted of a participant being randomly placed in an environment and having to navigate towards an object to collect it and to remember its location (mean duration 9.04 s per trial; SD = 3.12s). Participants had four trials to learn the location for each of the six objects interleaved in a set of 24 trials. Next participants were presented with a gray screen with a crosshair for 4 s between trials. After the learning phase was completed, there was a 30 s inter-phase rest period, when instructions on the next phase (object replacement phase) were presented. The object replacement phase for the location of each of the six collected objects started with a 3 s period in which an object was presented on a gray background (cue phase). Participants were then placed at a random location in the environment and told to navigate to the spot where they believed the pictured object had been located (mean duration = 12.41 s; SD = 4.14 s). They then pressed a button to indicate its previous location. Once the button was pressed, the ITI period would begin again for 4 s.

### Procedure and Design

Participants were instructed that they were going to navigate through a virtual environment over multiple sessions using a button box, and that they would have to pick up several different objects (six) in the environment, four times each (three objects, three times each for the two practice sessions). The order of trials was randomized (but unknown to participants) separated into three miniblocks. Object location never changed within a session. After they completed this learning phase, they were tested in an object replacement period, described above.

During the fMRI portion of the experiment, a new environment was presented and then represented at the next session as a familiar environment. This occurred on four occasions (three in the fMRI component), so that half of the eight environments were novel (refer to Fig. 1A). Each environment arena had the same area, but had its own unique shape (square, circle, triangle, and rectangle) and texture (desert, grass, snow, and rocky textures; refer to Fig. 1) to differentiate the environments.

Participants were presented with counterbalanced familiar or novel objects within each environment. Following the practice sessions, the objects presented in an environment were comprised of objects that the participants had either collected (“familiar”) or not collected (“novel”) in a previous session. Familiar objects were always from different environments in the fMRI experiment and the first familiar objects were introduced during the practice session outside of the scanner.

### fMRI Acquisition

Functional images were acquired on a 3T Siemens Trio scanner. Blood oxygenation level dependent (BOLD) T2*-weighted functional images were acquired using a gradient-echo EPI pulse sequence acquired obliquely at 45° with the following parameters: repetition time, 3,360 ms; echo time, 30 ms; slice thickness, 2 mm; interslice gap, 1 mm; in-plane resolution, 3 × 3 mm; field of view, 64 × 72 mm^2^; 48 slices per volume. A field-map using a double echo FLASH sequence was recorded for distortion correction of the acquired EPI (Weiskopf et al., 2006). After the functional scans, a T1-weighted 3-D MDEFT structural image (1 mm^3^) was acquired to co-register and display the functional data.

### fMRI Data Analysis

Functional images were processed and analyzed using SPM8 (www.fil.ion.ucl.uk/spm). The first five volumes were discarded to allow for T1 equilibration. Standard preprocessing included correction for differences in slice acquisition timing, realignment/unwarping to correct for interscan movement, and normalization of the images to an EPI template (specific to our sequence and scanner) that was aligned to the T1 MNI template. Finally, the normalized functional images were spatially smoothed with an isotropic 8 mm full-width half maximum Gaussian kernel. For all models, all regressors, with the exception of the movement parameters, were convolved with the SPM hemodynamic response function. Data were also high-pass filtered (cut-off period = 128 s).

Statistical analyses were performed using a general linear model within SPM8 with a block design for navigation periods during the learning phase, where we compared those navigation trial blocks to the blocks of adjacent intertrial intervals (ITI) to remove effects of slow variations in BOLD signal. Each fMRI session was modeled with seven regressors of interest, (1) navigation to novel objects during learning, (2) navigation to familiar objects during learning, (3) navigation with novel object cues during object replacement, (4) navigation with familiar object cues during object replacement, (5) novel object cue periods, and (6) familiar object cue periods, and (7) the ITI. Each trial was modeled as a boxcar function lasting the length of the navigation period (i.e., the length of time the participant took to “pick up” or “drop” the object for that specific trial). Although we explicitly modelled the cue periods, they were not used in our subsequent analyses, because of the low number and brief duration of trials. Each session included a further six “movement”' regressors estimated during realignment.

Six sessions were modeled, three relating to novel environments and three to familiar environments. This resulted in eight main conditions of interest (at both learning and object replacement; although objects were cued beforehand and not visible within an environment during object replacement): novel objects in a novel environment during the learning phase, familiar (previously seen) objects in a novel environment during the learning phase, novel objects in a familiar environment during the learning phase, familiar objects in a familiar environment during the learning phase, novel objects in a novel environment during the replacement phase, familiar objects in a novel environment during the replacement phase, novel objects in a familiar environment during the replacement phase, familiar objects in a familiar environment during the replacement phase. Each condition was contrasted with the session specific ITI prior to second-level analyses. From these conditions of interest, we ran an omnibus test to look at novelty effects during learning and replacement phases, which equated to a 2 × 2 × 2 (Experimental Phase × Environmental Novelty × Object Novelty) factorial design.

A further analysis split the learning phase into four quartiles (for each of the four object presentations, i.e., first–fourth presentations during the learning phase), to assess changes in novelty across encoding trials, resulting in 16 regressors (plus movement parameters). The six objects were repeatedly presented across four mini-blocks (first–fourth presentations), so that object presentation quartiles also coincided with the first quartile-fourth quartile of trials in a novel or familiar environment. Using a one-way ANOVA for first–fourth quartiles for each condition of interest, we investigated significant linear decreases over time for both novel versus familiar environments and objects.

Data were high-pass filtered (cut-off period = 128 s). Based on strong a priori hypotheses related to MTL involvement in novelty processing and use of a similar fMRI paradigm (Doeller et al., 2008,^2010^; Kaplan et al., 2012), we report activations surviving an uncorrected statistical threshold of *P =* 0.001 and cluster threshold *k* = 5 for the whole brain. Since we are using an uncorrected statistical threshold, we also report whether the peak voxel of MTL activations survive small-volume correction (SVC) for multiple comparisons (FWE p<.05) using a bilateral MTL mask encompassing the amygdala, hippocampus, and parahippocampal gyrus constructed in the automated anatomical labeling (AAL) toolbox for SPM (Tzourio-Mazoyer et al, 2002). Coordinates of brain regions are reported in MNI space. Post-hoc statistical analyses were conducted using 10 mm radius spheres in MarsBar (Brett et al., 2002) toolbox within SPM8 around the respective peak voxel specified in the corresponding results section to compare activity in one region across different conditions (e.g., to determine whether an object novelty effect was present in a region defined by the main effect of environmental novelty, or vice versa).

## RESULTS

### Behavioral Results

To assess behavioral performance, we looked at the distance error between where participants had indicated an object was during the object replacement phase and where it was actually located in the environment. Behavior was generally in line with past studies using this paradigm (Kaplan et al., 2012). The average length of navigation trials was 9.04 s (SD = 3.12 s) during the learning phase and 12.41 s (SD = 4.14 s) during the test phase. There were converse effects of environmental and object novelty on navigation time during the learning phase with more time spent navigating in novel versus familiar environments (*P* = 0.024; F(1,19) = 5.993) and less time spent navigating toward novel versus familiar objects (*P* = 0.01;F(1,19) = 8.272). However, there was no significant interaction between effects of object and environmental novelty on navigation times (*P* = 0.598; F(1,19) = 0.288). During the object replacement phase there were no significant differences in navigation trial times between novel versus familiar environments (*P* = 0.670; F(1,19) = 0.187), novel versus familiar objects (*P* = 0.395; F(1,19) = 0.758), or any interaction between effects of object and environmental novelty (*P* = 0.552; F(1,19) = 0.367). See Table1 for group means.

**Table 1:** Behavioral Data by Condition

	Novel object in a novel environment	Familiar object in a novel environment	Novel object in a familiar environment	Familiar object in a familiar environment
Mean learning phase navigation time (s)	9.49 (SD = 3.55)	10.3 (SD = 4.52)	7.96 (SD = 2.09)	8.47 (SD = 2.59)
Mean replacement phase navigation time (s)	12.19 (SD = 4.03)	12.86 (SD = 4.87)	12.26 (SD = 4.22)	12.36 (SD = 4.36)
Mean performance 1/distance error (virtual meters)	0.051 (SD = 0.020)	0.059 (0.025)	0.060 (SD = 0.025)	0.058 (SD = 0.024)

There were no significant performance differences between memory (i.e., 1/distance error) for novel versus familiar object locations (*P* = 0.268; F(1,19) = 1.31), or object locations in novel versus familiar environments (*P* = 0.281; F(1,19) = 1.23; see Table1 for group means). In line with our effect showing significant longer exploration durations for familiar versus novel objects, there was significantly enhanced performance for learning the location of novel versus familiar objects in novel environments (*P* = 0.049, *t*(19) = 2.11; see Table1 for group means). These findings are similar to previous behavioral findings showing proactive interference, where participants have impaired performance and need to spend more time learning object locations of “familiar” objects that were associated with a location in a previous environment (Kaplan et al., 2012). Over the course of the experiment, participants displayed a significant linear trend towards spending less time navigating during learning (*P* = 0.024; F(1,19) = 6.00) and test (*P* = 0.017; F(1,19) = 6.86) trials in later experimental sessions (see Table2 for group means). Participants also performed better in later sessions, exhibiting a marginal linear trend toward improved performance (increased 1/distance error) over sessions (*P* = 0.089; F(1,19) = 3.191; see Table2 for group means).

**Table 2:** Behavioral Data by Session

	fMRI session 1	fMRI session 2	fMRI session 3	fMRI session 4	fMRI session 5	fMRI session 6
Mean learning phase navigation time (s)	11.6 (SD = 7.96)	9.26 (SD = 3.57)	9.31 (SD = 4.11)	7.73 (SD = 2.23)	8.67 (SD = 3.10)	7.66 (SD = 2.90)
Mean replacement phase navigation time (s)	13.4 (SD = 5.02)	13.6 (SD = 6.13)	12.5 (SD = 3.88)	12.0 (SD = 4.52)	11.7 (SD = 4.64)	11.3 (SD = 4.78)
Mean performance 1/distance error (virtual meters)	0.049 (SD = 0.0262)	0.056 (SD = 0.0261)	0.056 (SD = 0.0270)	0.054 (SD = 0.0268)	0.060 (SD = 0.0326)	0.067 (SD = 0.0299)

### fMRI Results

#### Environmental novelty

**Figure 2:**
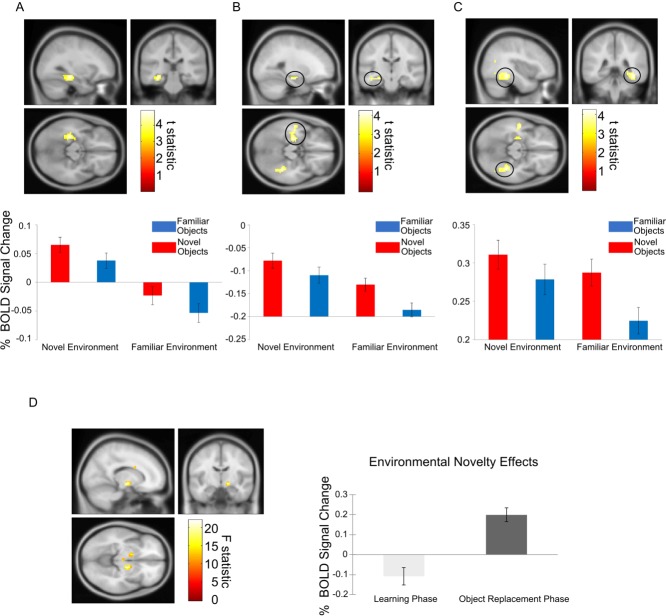
Environmental and object novelty during the navigation task. A: Left hippocampal activity corresponding to environmental novelty during navigation (above; peak voxel: *x* = −30; *y* = −28; *z* = −14; *Z*-score = 4.65; including both learning and object replacement phases). Percent signal change in a 10 mm sphere around the left hippocampal peak for all four conditions (navigating toward or replacing novel or familiar objects within novel or familiar environments, below, showing mean ± SEM over the 18 participants). B: Left anterior hippocampal activity, extending into the amygdala and superior temporal sulcus, corresponding to object novelty during navigation (above, peak voxel: *x* = 39; *y* = −40; *z* = −14; *Z*-score = 3.81). Percent signal change in a 10 mm sphere around the left anterior hippocampal peak for all four conditions (below, mean ± SEM). C: Left parahippocampal/fusiform activity corresponding to object novelty during navigation (above, peak voxel: *x* = 39; *y* = −40; *z* = −14; *Z*-score = 4.10). Percent signal change in a 10 mm sphere around the left parahippocampal/fusiform peak for all four conditions (mean ± SEM). D: Right ventral pallidum activity corresponding to the interaction between environmental novelty and experimental phase (left; peak voxel: *x* = 15; *y* = −10; *z* = −8; *Z*-score = 4.34; left ventral pallidum and midbrain/VTA effects visible in axial slice). Percent signal change in a 10 mm sphere around the right ventral pallidal peak during navigation in novel versus familiar environments during the learning and replacement phases (mean ± SEM). All activations are shown at the uncorrected threshold of *P* < 0.001 for display purposes and overlaid on the Montreal Neurological Institute 152 T1 image. [Color figure can be viewed in the online issue, which is available at wileyonlinelibrary.com.]

We used a 2 × 2 × 2 ANOVA (Object × Environment × Phase) to test whether there were significant differences for object and environmental novelty processing during learning and object replacement phases. When contrasting navigation in novel versus familiar environments (collapsed across learning and replacement phases), the strongest increase across the whole brain was in the medial temporal lobe, with a peak in the left posterior hippocampus/parahippocampal gyrus (*x* = −30; *y* = −28; *z* = −14; *Z*-score = 4.65; SVC FWE *P* = 0.001; see Fig. 2A and Table3) and another subpeak that was part of the same cluster in the anterior hippocampus (*x* = −27; *y* = −19; *z* = −17; *Z*-score = 3.56).

**Table 3:** Main Effect of Environmental Novelty

Region	*x*	*y*	*z*	Z-score
L Hippocampus/Parahippocampal Gyrus	−30	−28	−14	4.65
L Cerebellum	−15	−52	−38	3.58
L Dorsolateral Prefrontal Cortex	−54	41	4	3.56
L Angular Gyrus	−66	−49	1	3.52
R Precuneus	27	−79	16	3.41
R Superior Parietal Lobule	48	−73	13	3.40
R Caudate	18	17	19	3.26

In a post-hoc statistical analysis based on 10 mm spheres around the peak hippocampal voxel for environmental novelty, the left posterior hippocampus cluster also responded to object novelty (*F* = 7.584; *P* = 0.014), but no interaction between environmental and object novelty was observed (*F* = 0.019; *P* = 0.892). Despite our posterior hippocampal peak also responding to object novelty, we found that the posterior hippocampus showed a significantly stronger response to environmental novelty than to object novelty [t(17) = 3.350; *P* = 0.004]. We also found increases related to environmental novelty in the left cerebellum, left dorsolateral prefrontal cortex, left angular gyrus, right precuneus, right superior parietal lobule, and right caudate (see Table3). Additionally, subthreshold right hippocampal activations for environmental novelty were observed at *P* < 0.005 uncorrected. A post-hoc paired t-test conducted on data extracted from 10 mm spheres around left and right hippocampus peak voxels did not reveal a significant difference between the left and right hippocampus in their response to environmental novelty [t(17) = 1.515; *P* = 0.148]. There were no significant increases in the brain for environmental familiarity.

#### Object novelty

We found a main effect for novel versus familiar objects, regardless of environment, in the bilateral parahippocampal/fusiform gyrus that was strongest in the right hemisphere (right peak: *x* = 39;*y* = −40; *z* = −14; *Z*-score = 4.10; whole-brain cluster-level FWE *P* = 0.002; see Fig. 2C and Table4). There was also a significant MTL cluster in the left anterior hippocampus (*x* = −24; *y* = −19; *z* = −17; *Z*-score = 3.81; SVC FWE *P* = 0.032; see Fig. 2B and Table4), which extended into the amygdala and left superior temporal sulcus. Post-hoc statistical analyses measured from a 10 mm sphere around the posterior parahippocampal/fusiform gyrus peak found that it was not sensitive to environmental novelty (*F* = 1.991, *P* = 0.176) and showed no interaction between object and environmental novelty effects (*F* = 1.505; *P* = 0.237). Notably, the anterior hippocampal peak overlapped with the subpeak from the environmental novelty contrast, which was reflected in post-hoc statistical analyses that showed that the anterior hippocampus was also sensitive to environmental novelty (*F* = 6.486; *P* = 0.021), but displayed no interaction between the two novelty effects. Further analyses showed that the anterior hippocampus was not differentially responsive to object novelty or environmental novelty [*t*(17) = 0.766; *P* = 0.454]. We also found significant increases related to object novelty in the cingulate gyrus and right angular gyrus (see Table4). There were no significant increases anywhere in the brain for object familiarity.

**Table 4:** Main Effect of Object Novelty

Region	*x*	*y*	*z*	Z-score
R Parahippocampal/Fusiform Gyrus	39	−40	−14	4.10
L Anterior Hippocampus/Amygdala/Superior Temporal Sulcus	−24	−19	−17	3.81
L Parahippocampal/Fusiform Gyrus	−30	−49	−8	3.77
Cingulate Gyrus	12	−4	40	3.59
R Angular Gyrus	39	−64	19	3.47

### Novelty × Experimental Phase Interactions

We did not find any significant interactions in the medial temporal lobe between object novelty and experimental phase, or between environmental novelty and experimental phase. In other words, the MTL object and environmental novelty effects did not differ as a function of learning versus replacement phase. However, an environmental novelty by experimental phase interaction was seen in bilateral ventral pallidum, strongest on the right side (left: *x* = −15; *y* = −1; *z* = −11; *Z*-score = 3.56; right: *x* = 15; *y* = −10; *z* = −8; *Z*-score = 4.34; see Fig. 2D). We also found a significant interaction between environmental novelty and experimental phase in the midbrain/ventral tegmental area (VTA; *x* = 3; *y* = −19; *z* = −5; *Z*-score = 3.57) and right caudate. Notably, environmental novelty related activity was higher in the replacement versus the learning phase in the ventral pallidum and midbrain/VTA, while the caudate showed the opposite effect of being higher in the learning phase than the replacement phase. Additionally, we observed a significant interaction between object novelty and experimental phase in the cerebellum and cingulate gyrus, where object novelty related increases during navigation were higher in the object replacement versus learning phase.

### Short-Term Effects of Novelty During Learning

**Figure 3:**
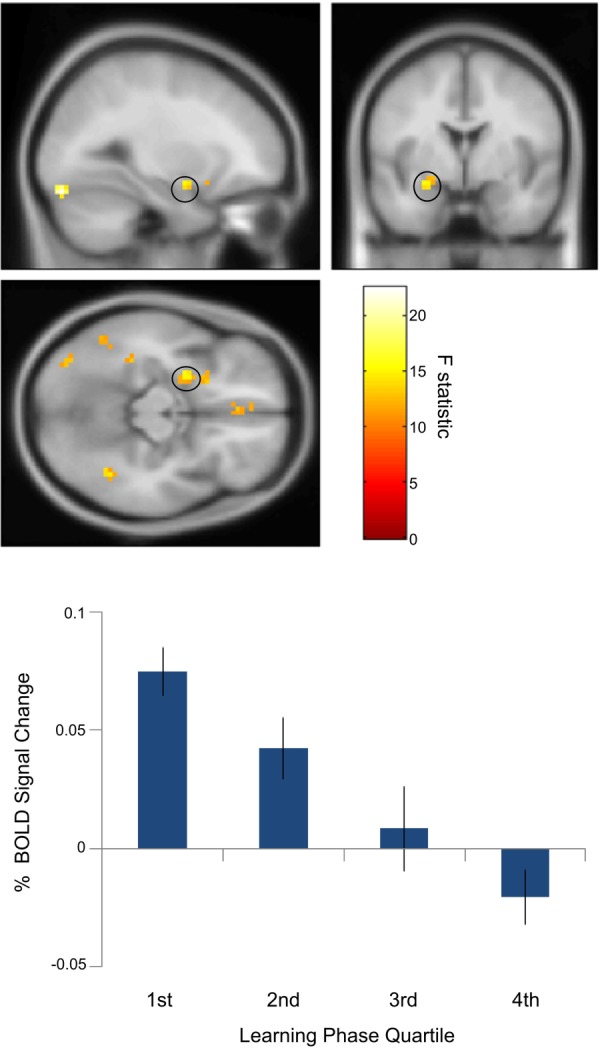
Temporal attenuation of amygdala object novelty effect. Above: Left amygdala activity (peak: *x* = −27; *y* = −1; *z* = −14; *Z*-score = 3.65) corresponding object novelty (novel versus familiar objects) showed a linear decrease over the course of the learning phase as relative novelty decreased. Below: Percent signal change extracted from a 10 mm sphere around the peak left amygdala voxel averaged across 18 participants for novel versus familiar objects for the first to fourth quartile of the learning phase. Activations are shown at the uncorrected threshold of *P* < 0.001 and overlaid on the Montreal Neurological Institute 152 T1 image for display purposes. [Color figure can be viewed in the online issue, which is available at wileyonlinelibrary.com.]

We investigated how the above environmental and object novelty effects changed across time during the learning phase. We split learning phases into four quartiles (matching the four repetitions of object-location encoding during the learning phase) and assessed both environmental and object novelty across these quartiles. Searching for a linear decrease of environmental novelty from quartiles 1–4 failed to reveal any significant regions in the MTL or neocortex. However, a significant linear decrease in the object novelty effect (i.e., novel – familiar) from the first to fourth quartile was seen in the left amygdala (peak: *x* = −27; *y* = −1; *z* = −14; *Z*-score = 3.65; uncorrected *P* = 0.000131; FWE SVC *P* =.074), left lateral occipital cortex (peak: *x* = −30; *y* = −85; *z* = −20; *Z*-score = 4.50), left fusiform gyrus (peak: *x* = −48; *y* = −55; *z* = −17; *Z*-score = 3.74), and left posterior parahippocampal cortex (peak: *x* = −36; *y* = −37; *z* = −17; *Z*-score = 3.87). See Figure 3. Other areas showing a significant decrease in the object novelty effect from the first to fourth quartile were the bilateral ventrolateral prefrontal cortex, ventromedial prefrontal cortex, left middle temporal gyrus, and right angular gyrus. These analyses add to our original object novelty effects (comparing whole sessions) by revealing several novelty signals that attenuate within each session. By contrast, environmental novelty signals appear to attenuate only over the slower timescale of sessions, perhaps indicating that learning environmental layout is a slower process than some of the short-term effects of object novelty.

## DISCUSSION

We investigated environmental and object novelty during a spatial navigation task. We found environmental and object novelty effects throughout the MTL, including the hippocampus (Fig. 2). Environmental novelty effects were seen in the mid-posterior hippocampus, anterior hippocampus, and parahippocampal gyrus. Object novelty effects were seen in the anterior hippocampus, fusiform/parahippocampal gyrus, and amygdala. Notably, the more posterior hippocampal region showed a greater response to environmental than object novelty, while the anterior hippocampus peak responded to both environmental and object novelty. Thus, we provide evidence for distinct, though partially overlapping, MTL networks for processing environmental and object novelty.

Our finding that the anterior hippocampus responds to both object novelty and environmental novelty is consistent with previous fMRI studies showing anterior hippocampal responses to the novelty of a variety of stimuli (Stern et al., 1996; Pihlajamaki et al., 2004; Kohler et al., 2005; Strange et al., 2005a,b; Kumaran and Maguire, 2006). To our knowledge, our findings are the first to show responses to both environmental and object novelty during goal-directed virtual navigation. However, our results also suggest a partial dissociation within the hippocampus, with more posterior hippocampal regions showing a greater response to environmental novelty than to object novelty. Mid-posterior hippocampal activation related to environmental novelty is consistent with a role in learning environmental layout, including the spatial relations between the various topographical features of the environment (the environmental boundary and the distant mountains). This fits with the known role of the hippocampus in encoding spatial layout (e.g., Lee et al., 2005; Hartley et al., 2007) and when navigating more accurately (Maguire et al., 1998; Hartley et al., 2003). The findings are also consistent with a more general hippocampal role in representing relational information (Eichenbaum and Cohen, 2001) and with rodent studies showing hippocampal involvement in detecting environmental novelty (Save et al., 1992a, b; Moses et al., 2002, 2005; Lee et al., 2005; Good et al., 2007). An important potential research direction will be determining whether the anterior hippocampus might function as a convergence zone (Damasio, 1989) for processing both object/item and environmental/contextual novelty (Gaffan, 1998; Diana et al., 2007; Eichenbaum et al., 2007; Ranganath and Ritchey, 2012) and how these hippocampal processing distinctions relate to ideas about “nonspatial” and “spatial” processing streams within the MTL (Knierim et al., 2006). Notably, we did not find any novelty processing differences between learning and object replacement phases in the hippocampus, or the rest of MTL. Future studies can explore how hippocampal sub-regions might process novelty differently depending on whether a subregion needs to either encode or retrieve an item/context.

Outside of the hippocampus, we found that the parahippocampal/fusiform gyrus responded to object novelty and that more anterior parts of the parahippocampal gyrus responded to environmental novelty. Our finding of parahippocampal responses to environmental novelty is in line with previous fMRI findings showing that the parahippocampal cortex responds to novel scenes (Epstein and Kanwisher, 1998; Epstein et al., 2003). However, our activation was slightly anterior to the “place area” regions typically associated with the perceptual processing of environmental scenes, and so may relate more specifically to the learning of environmental layout for the purposes of navigation, and the nearby activation of the hippocampus. In fact, our object novelty activation was located in these more posterior parahippocampal/fusiform regions. We assume that this activation reflects the introduction of a novel navigationally relevant object into posterior parahippocampal representations of the environment (Janzen and van Turennout, 2004; Turk-Browne et al., 2012). That is, the effect of object novelty in this region reflects the fact that the objects themselves are navigationally relevant components of the wider spatial environment. Nonetheless, fusiform regions are also thought to be involved in representing individual objects (Grill-Spector et al., 2001; Ewbank et al., 2005; Horner and Henson, 2008), so the fusiform object-novelty activation may also reflect the learning of novel object representations.

We also found effects of object novelty in the amygdala. These results parallel previous fMRI findings showing amygdala and anterior hippocampal responses to novelty (Blackford et al., 2010; Bunzeck et al., 2010; Balderston et al., 2011; Thoresen et al., 2012). They also accord with rodent studies showing that amygdala lesions impair object novelty detection (Moses et al., 2002, 2005), and with human intracranial recordings showing amygdala (and sometimes anterior hippocampal) responses to novel or surprising events (Halgren et al., 1980; Fried et al., 1997; Rutishauser et al., 2010). The amygdala activity during learning was characterized by a linear reduction in response to novel objects across repetitions, with the maximal effect for the first presentation of the object. This rapidly decaying amygdala activation might reflect a temporary novelty-related increase in salience as opposed to our novelty responses over the longer timescale of trials, which we tentatively related to learning. Rapidly attenuating amygdala activations have also been seen in response to emotional/arousing stimuli (Breiter et al., 1996; Fischer et al., 2003; Schwartz et al., 2003; Manelis et al., 2012).

One caveat for the interpretation of our findings is that we only studied males, to avoid compromising our result with the additional uncontrolled variable of the sexual dimorphism in neural bases of spatial navigation (Gron et al., 2000; see Maguire et al., 1999 and Driscoll et al., 2005 for reviews). Gender differences could especially be important for the amygdala and anterior hippocampus results, since hormonal release potentially modulates behavior in these areas during mnemonic function (Strange and Dolan, 2006). Further study will be needed to see if our findings generalize to females, or whether they differ between the sexes. And further replication will be required to corroborate the rapidly attenuating amygdala object novelty effect during the learning phase, which did not pass correction for multiple comparisons.

Although our MTL novelty effects did not differ across the learning and replacement phases, we did observe that ventral pallidum and midbrain/VTA BOLD activity was higher during navigation in novel versus familiar environments during the replacement phase, while environmental novelty related activity in the caudate was higher during the learning phase. The ventral pallidum and midbrain/VTA effects are consistent with findings highlighting strong responses in ventral basal ganglia and midbrain/VTA regions for images of novel versus familiar scenes (Guitart-Masip et al., 2010). Our ventral pallidum findings match rodent studies showing this region as a key interchange between limbic and movement-planning circuitry that helps guide goal-directed exploratory movement (Yang and Mogenson, 1985; for review see Mogenson and Yang, 1991), while our caudate findings might relate to the formation of more route-like representations from one object location to another as their locations are learned, consistent with the parallel hippocampal and striatal involvement in the learning of “places” and “responses” (e.g., Packard and McGaugh, 1996; Iaria et al., 2003; White and McDonald, 2002; Hartley et al., 2003; Voermans et al., 2004). Although further replication is needed, our results might reflect a potential role for interactions between the MTL and basal ganglia in guiding the memory of novel contexts (see reviews by Pennartz et al., 2011; van der Meer et al., 2012) and in disambiguating overlapping routes (Brown et al., 2012; Brown and Stern, 2014).

## CONCLUSION

We employed a naturalistic virtual reality navigation paradigm to assess how the human brain processes novel environments and their contents (i.e., object-location associations). We found that the anterior hippocampus responded to both environmental and object novelty during navigation, whereas mid to posterior hippocampus preferentially responded to environmental novelty, consistent with a role in representing the layout of a new environment. Our results suggest that the MTL is crucial in processing both object and environmental novelty during spatial navigation and that novelty processing is likely to be supported by a distinct, but partially overlapping, set of regions in the MTL.
